# The Neurotransmitter Receptor Architecture of the Mouse Olfactory System

**DOI:** 10.3389/fnana.2021.632549

**Published:** 2021-04-23

**Authors:** Kimberley Lothmann, Katrin Amunts, Christina Herold

**Affiliations:** ^1^C. & O. Vogt-Institute of Brain Research, Medical Faculty, University Hospital Düsseldorf, Heinrich Heine University of Düsseldorf, Düsseldorf, Germany; ^2^Institute of Neuroscience and Medicine INM-1, Research Centre Jülich, Jülich, Germany

**Keywords:** receptor autoradiography, rodent, neurotransmitter receptors, olfactory cortex, taenia tecta, dorsal peduncular cortex, entorhinal cortex, orbitofrontal cortex

## Abstract

The uptake, transmission and processing of sensory olfactory information is modulated by inhibitory and excitatory receptors in the olfactory system. Previous studies have focused on the function of individual receptors in distinct brain areas, but the receptor architecture of the whole system remains unclear. Here, we analyzed the receptor profiles of the whole olfactory system of adult male mice. We examined the distribution patterns of glutamatergic (AMPA, kainate, mGlu_2/3_, and NMDA), GABAergic (GABA_A_, GABA_A(BZ)_, and GABA_B_), dopaminergic (D_1/5_) and noradrenergic (α_1_ and α_2_) neurotransmitter receptors by quantitative *in vitro* receptor autoradiography combined with an analysis of the cyto- and myelo-architecture. We observed that each subarea of the olfactory system is characterized by individual densities of distinct neurotransmitter receptor types, leading to a region- and layer-specific receptor profile. Thereby, the investigated receptors in the respective areas and strata showed a heterogeneous expression. Generally, we detected high densities of mGlu_2/3_Rs, GABA_A(BZ)_Rs and GABA_B_Rs. Noradrenergic receptors revealed a highly heterogenic distribution, while the dopaminergic receptor D_1/5_ displayed low concentrations, except in the olfactory tubercle and the dorsal endopiriform nucleus. The similarities and dissimilarities of the area-specific multireceptor profiles were analyzed by a hierarchical cluster analysis. A three-cluster solution was found that divided the areas into the (1) olfactory relay stations (main and accessory olfactory bulb), (2) the olfactory cortex (anterior olfactory cortex, dorsal peduncular cortex, taenia tecta, piriform cortex, endopiriform nucleus, entorhinal cortex, orbitofrontal cortex) and the (3) olfactory tubercle, constituting its own cluster. The multimodal receptor-architectonic analysis of each component of the olfactory system provides new insights into its neurochemical organization and future possibilities for pharmaceutic targeting.

## Introduction

Olfactory areas form a highly interconnected network to process chemical olfactory information for olfactory recognition, memory, mating, and learning in mice. Thereby the olfactory system consists of two main subdivisions, the main and the accessory olfactory system. The processing of sensory stimuli into functional olfactory information is further accomplished by the primary and the secondary olfactory cortex. The primary olfactory cortex is composed of the anterior olfactory cortex (anterior olfactory nucleus), the taenia tecta (dorsal, ventral), the dorsal peduncular cortex, the olfactory tubercle, the piriform cortex with the dorsal endopiriform nucleus and the entorhinal cortex (lateral and medial). These structures directly receive input from the main olfactory bulb and project back (Luskin and Price, 1983; Shepherd, 2004; Wilson et al., 2015). The orbitofrontal cortex (medial, lateral, and ventrolateral), particularly the medial part, represents the secondary olfactory cortex. While the peripheral sensory organs of the olfactory epithelium and vomeronasal organ initiate the uptake, different neurotransmitter systems of the main and accessory olfactory CNS serve to process the information and, moreover, modulate cortical and subcortical olfactory areas via feedback-regulation. Therefore, we focused on all known cortical and subcortical olfactory areas and their receptor architecture in the mouse brain.

Sensory information is first processed in the main and accessory olfactory bulb via various neurotransmitters, either supplied directly by local interneurons of both, the main and accessory olfactory bulbs (main olfactory bulb: Murphy et al., 2004; Shepherd, 2004; Ennis et al., 2007; Linster and Cleland, 2016; Blakemore et al., 2018; Dong and Ennis, 2018; accessory olfactory bulb: Brennan et al., 1995; Dudley and Moss, 1995; Jia et al., 1999; Mohrhardt et al., 2018), via the horizontal limb of the diagonal band (GABAergic and cholinergic input; Brashear et al., 1986; Zaborszky et al., 1986) and the locus coeruleus (noradrenergic input; McLean et al., 1989). Several studies focused on the role of neurotransmitters in the olfactory system and showed that AMPARs/kainateRs and NMDARs regulate the excitability of mitral and tufted cells (Salin et al., 2001; Urban and Sakmann, 2002; Christie and Westbrook, 2006; Blakemore et al., 2018). Further, glutamatergic receptors play a role in vomeronasal stimulation in the glomeruli of the accessory olfactory bulb, enabling social and reproductive events (Dudley and Moss, 1995; Mohrhardt et al., 2018). Group II metabotropic glutamate receptors (mGluRs) (Zak et al., 2015), GABAergic GABA_A_Rs and GABA_B_Rs (Smith and Jahr, 2002; Shepherd, 2004; Panzanelli et al., 2005) enable the contrasting of odors, while GABA_A_ benzodiazepine binding sites (GABA_A(BZ)_Rs) play a pivotal role in olfactory discrimination learning (McGregor et al., 2004; Sokolic and McGregor, 2007). Noradrenaline receptors are active during reward odor responses (Doucette et al., 2011), while dopamine receptors generate odor-preference and modulate reward signals in the olfactory tubercle (Zhang et al., 2017).

However, previous studies that examined the receptor distribution regarding specific functions of olfactory areas often focused on larger areas, for example, the main olfactory bulb (Shepherd, 2004; Ennis et al., 2007), the piriform (Petralia and Wenthold, 1992; Wisden and Seeburg, 1993; Petralia et al., 1994a,b; Wada et al., 1998; Ennis et al., 2007) and entorhinal cortex (Fotuhi et al., 1994; Caruana et al., 2006; Thompson et al., 2006; West et al., 2007; Middleton et al., 2008; Glovaci et al., 2014; Glovaci and Chapman, 2019), while the smaller areas like the taenia tecta, the dorsal peduncular cortex and the dorsal endopiriform nucleus were less covered. Furthermore, most studies focused on a specific receptor or receptor family. For example, there have been studies (for review see: Shepherd, 2004; Ennis et al., 2007) providing information about the receptors in individual olfactory areas, but no reference atlas of the entire system has been generated to provide a basis to link the accumulated knowledge on a chemoarchitectural level.

Up to now, the cytoarchitecture, the connectivity and functionality of the rodent olfactory areas are widely known (Dong, 2008; Ennis et al., 2008, 2015; Mucignat-Caretta, 2010; Franklin and Paxinos, 2013; Cleland and Linster, 2019), with exception of the taenia tecta and the dorsal peduncular cortex. The taenia tecta is divided into two parts. The dorsal part is referred to the hippocampal formation while the ventral part corresponds to the main olfactory cortex (Haberly and Price, 1978; Luskin and Price, 1983; Santiago and Shammah-Lagnado, 2005; Shiotani et al., 2020). To gain a more comprehensive understanding of the olfactory system including its different areas, it is necessary to focus on these regions as well because they are part of the primary olfactory cortex and connect the olfactory network within the limbic system (Dong, 2008; Ennis et al., 2008; Mucignat-Caretta, 2010; Franklin and Paxinos, 2013; Ennis et al., 2015; Cleland and Linster, 2019).

In addition, the *in vitro* receptor autoradiography of the olfactory system shows anatomically distinct and different receptor densities at a high spatial resolution. This provides a basis for the validation/development of existing/potential positron emission tomography (PET) tracers, a technique with a 10-times lower resolution compared to autoradiography (Bergström et al., 2003). PET as an interdisciplinary, non-invasive *in vivo* method is used for the study of neuropharmacological drug-receptor targets, particularly in rodent brains (Lancelot and Zimmer, 2010; Herfert et al., 2020). Importantly, the olfactory system seems to play a pivotal role in the neuropathology of neurodegenerative diseases like Alzheimer's or Parkinson's disease. Here, olfactory dysfunction is one of the first clinical symptoms (Mesholam et al., 1998; Albers et al., 2006; Zou et al., 2016). Thus, the understanding of the specific receptor distribution may help to find strategies for new therapeutic targets. Therefore, we investigated the receptor distribution of glutamatergic (AMPA, kainate, NMDA, mGlu_2/3_), GABAergic (GABA_A_, GABA_A(BZ)_, GABA_B_), noradrenergic (α_1_, α_2_) and dopaminergic (D_1/5_) receptors in detail. Our findings not only resulted in a chemoarchitectonic parceling of the olfactory areas and layers, but also provides a detailed, layer-specific multi-receptor profile of all known associated regions of the entire olfactory system. The comparative analysis of the different receptor profiles (ratio of the different receptors in a brain area) within the system may also help to gain further insights into hitherto undiscovered area functionality. Therefore, the specific analysis of multiple receptor expressions of the entire rodent olfactory system and the observed receptor architecture can serve as a comparative multireceptor map and provides further insights in previously less studied regions such as the taenia tecta and the dorsal peduncular cortex. We also provide a molecular organization profile within the primary olfactory cortex by multi-receptor fingerprints and a subsequent multidimensional cluster analysis.

## Materials and Methods

### Animals and Tissue Preparation

Ten, adult male C57BL/6 mice from CERJ (Janvier Labs, Germany) were kept in two groups (five animals/cage) in an enriched environment under constant room temperature and humidity control in a 12-h light-dark cycle for 8 weeks. Water and food pellets were provided *ad libitum*. At the time of brain removal, each mouse was 27 weeks in age.

The project was implemented in accordance with the guidelines of the “Landesamt für Natur, Umwelt und Verbraucherschutz NRW, Germany (LANUV),” the directives of the National Institute of Health Guide for Care and Use of Laboratory Animals in addition to the German Animal Welfare Act (Az.87-51.04.2010.A250).

To perform the histology and receptor autoradiography the ten mice were decapitated, and their brains were directly removed and frozen in isopentane at −40°C. Until dissection, the tissues were stored at −80°C and were processed contemporary.

### Histology and Receptor Autoradiography

Serial coronal sections (16 μm) were obtained from a hemisphere/animal (in total *n* = 10, left and right hemispheres were randomized) using a cryostat microtome (Leica, Germany). Each brain was used for all ligands. Overall, a total of 37–40 slices were processed from a single mouse brain for each ligand. For the analysis of the olfactory system, only sections between Bregma level 3.56 to −2.92 mm, previously identified using a cytoarchitectonic atlas of the C57BL6 mouse brain (Hof et al., 2000), were analyzed. To achieve a sufficient number of data per region of interest, at least nine slices per ligand and mouse were used. This gave us a total of 90 sections per mouse for receptor autoradiography and 18 sections per mouse for histology (cell body and myelin staining). After dissecting, slices were frozen and stored at −80° to process all slices for each ligand at the same time. The detailed autoradiographic labeling procedure has been published elsewhere (Zilles et al., 2002a,b; Schleicher et al., 2005). The glass-mounted slices have been prepared according to a standard protocol for quantitative *in vitro* receptor autoradiography (Zilles et al., 2002a,b; Palomero-Gallagher et al., 2009; Herold et al., 2017; Supplementary Table 1). Glutamatergic, GABAergic, noradrenergic und dopaminergic receptors were labeled using the respective tritiated ligands: [^3^H]AMPA (AMPAR), [^3^H]kainate (kainateR), [^3^H]MK-801 (NMDAR), [^3^H]LY-341495 (mGlu_2/3_R), [^3^H]muscimol (GABA_A_R), [^3^H]flumazenil (GABA_A_ associated benzodiazepine (BZ) binding sites, GABA_A(BZ)_R), [^3^H]CGP-54626 (GABA_B_R), [^3^H]prazosin (α_1_R), [^3^H]RX-821002 (α_2_R), and [^3^H]SCH-23390 (D_1/5_R). Additionally, cryosections were stained for cell bodies in regular intervals using standard staining protocols to label cell bodies and myelinated fibers for cyto- and myeloarchitectonic analysis, respectively (Gallyas, 1971; Merker, 1983).

The first step of receptor autoradiography involved the pre-incubation of the sections by rinsing the endogenous ligand. Subsequently, the main incubation is performed. Here, both the total binding as well as the unspecific binding is determined. For total binding, sections were labeled with the tritiated receptor ligand, whereas in unspecific binding, a specific, unlabeled displacer was co-incubated. To stop the binding process, slices were rinsed with buffer to remove the non-specific radioactive ligand. Thereafter, the radioactively marked tissue sections were exposed to tritium-sensitive hyperfilms (Hyperfilm, Amersham, UK) for 10–18 weeks together with isotope [^3^H]-standards of known radioactivity concentrations (Microscales, Amersham, UK). The exposure time depends on the binding saturation. The time has to be sufficiently long for measurable signals, but not too long to obtain a good signal-to-noise ratio and a low background signal. All slices of one brain hemisphere were simultaneously and constantly exposed: 10 weeks (GABA_A(BZ)_), 12 weeks (kainate, NMDA, mGlu_2/3_, GABA_A_, GABA_B_), or 15 weeks (AMPA, D_1/5_).

### Image Analysis

**Figure 1:**
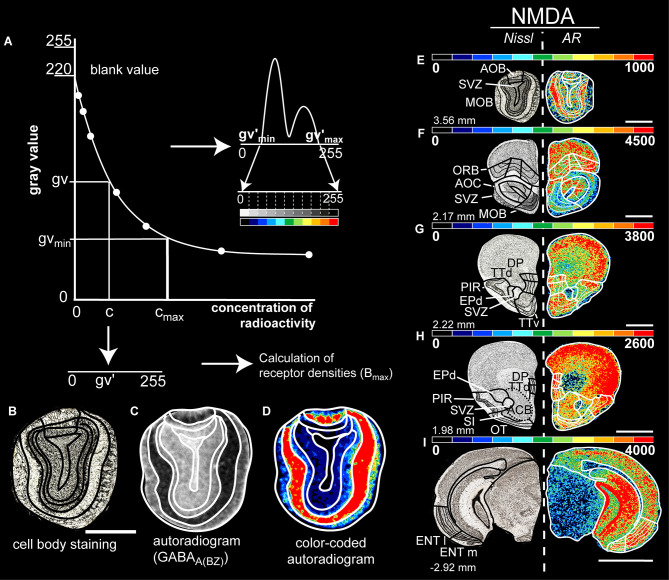
**(A)** Calibration curve of the co-exposed isotope standards of known radioactivity for computation of the concentration of the bound ligand. A non-linear dependence of gray values and radioactivity concentration is provided. This allows the gray values of the autoradiograms to be converted pixel by pixel to the corresponding concentration of radioactivity. A gray level histogram of the transformed autoradiogram **(C)** was generated, followed by a linear contrast enhancement procedure. Here, the gray-scale histogram is transformed into a spectral arrangement of 11 colors to facilitate visualization of the density patterns on the autoradiograms **(D)**. A brain section of the same level with cell body staining **(B)** is used simultaneously to determine the boundaries. Figure **(A)** adapted by Zilles and Schleicher (1995) and Herold et al. (2014). (**E–I)** Exemplary figure of the border identification in color-coded images that shows the distribution and density of glutamatergic NMDA receptors at different Bregma levels (left). The color scale of 11 equally distributed colors corresponds to the densities of receptors in fmol/mg protein. The color scale of each image has been optimized to provide the best visualization for different densities of the receptors. Therefore, the red end corresponds to the best visual fit for the investigated olfactory structure that is not necessarily the maximum density. Scale bars: **(B–E)**, 1.3 mm; **(F–H)**, 1.5 mm; **(I)**, 2 mm; MOB, main olfactory bulb; AOB, accessory olfactory bulb; AOC, anterior olfactory nucleus; TTd, taenia tecta, dorsal; TTv, taenia tecta, ventral; DP, dorsal peduncular cortex; PIR, piriform cortex; EPd, dorsal endopiriform nucleus; ENTl, entorhinal cortex, lateral; ENTm, entorhinal cortex, medial; ORB, orbitofrontal cortex; OT, olfactory tubercle; SVZ, subependymal zone; SI, substantia innominate.

The resulting autoradiographs were densiometrically processed with an image analysis technique (Figure 1, Zilles et al., 2002b, Schleicher et al., 2005). They were digitized using AxioVision Rel. 4.7 (Zeiss, Germany), in-house scripts and an AxioCam camera (Zeiss, Japan). The images were stored as binary files with a resolution of 512 × 512 pixels and 8-bit gray value. Initially, a calibration curve (Figure 1A) was defined using the gray values of the microscales with known radioactivity concentration. According to the specific experimental conditions, this calibration curve was adjusted specifically for each ligand (e.g., specific activity, dissociation constant, and free concentration of the ligand during incubation; see Zilles et al., 2002b; Zilles and Palomero-Gallagher, 2017). The concentrations of binding sites were transformed into fmol/mg protein at saturation conditions by

(1) $$\begin{array}{l} B_{\max}=\;\frac{K_{D}+L}{A_{S}\times L} \end{array}$$

where K_D_ is the equilibrium dissociation constant of ligand-binding kinetics, L the incubation concentration of ligand, A_S_ the specific activity of the ligand. The results determine the binding site density measurements.

The calibration curve is used to compute the gray values of autoradiography images into their corresponding concentrations of radioactivity. By non-linear, least-squares fitting, the relationship between gray values of the autoradiographs and concentrations of radioactivity is defined. By interpolation of each pixel into the calibration curve, the pixel is converted into the corresponding concentration of radioactivity. Subsequently, it is linearly transformed into a new range of 256 gray values (0, black to 255, white) to create a linearized autoradiogram in which gray values are a linear function of the concentration of radioactivity (Figure 1A).

### Anatomical Identification

For the identification of the regions of interest we converted the original autoradiogram into its pseudo-color-coded ligand-specific image for visual purposes (Figures 1C,D). Based on a predefined spectral assignment of 11 colors to the density ranges, an optimized visualization of the densities of the autoradiograms is achieved (Figures 1E–I; Zilles and Schleicher, 1995).

Subsequently, we compared these colored autoradiograms with the adjacent Nissl or myelin sections, the brain atlas of Paxinos (Franklin and Paxinos, 2013) and the Allen Brain Atlas (Lein et al., 2007; Dong, 2008) to locate the boundaries of the brain regions (Figures 1B–D).

The obtained receptor densities of each investigated region of the olfactory system were calculated over all available tissues of a hemisphere for each animal, averaged over the ten animals and given as total receptor concentration (mean concentration ± standard error of means in fmol/mg protein; Table 1, Supplementary Tables 2, 3). The quantitative multi-receptor data are presented in color-coded autoradiographs (**Figures 3**–**9**), a heat map (**Figure 10**) and regional receptor fingerprints (**Figure 11**).

**Table 1:** Neurotransmitter receptor densities (fmol/mg protein) in different regions of the mouse olfactory system (Mean ± SEM).

	**Receptor (fmol/mg protein)**										
	**AMPA**	**Kainate**	**NMDA**	**NMDA**	**mGlu_**2/3**_**	**GABA_**A**_**	**GABA_**A(BZ)**_**	**GABA_**B**_**	**α_1_**	**α_2_**	**D_**1/5**_**
Main olfactory bulb	727 ± 54	1,281 ± 95	954 ± 158	954 ± 158	2,180 ± 121	803 ± 93	3,867 ± 577	2,144 ± 119	547 ± 33	497 ± 32	61 ± 6
Accessory olfactory bulb	909 ± 88	1,328 ± 94	1,203 ± 156	1,203 ± 156	3,948 ± 376	950 ± 212	6,409 ± 664	3,617 ± 288	675 ± 50	547 ± 69	100 ± 15
Anterior olfactory cortex	1,044 ± 87	1,432 ± 69	1,445 ± 189	1,445 ± 189	2,365 ± 165	566 ± 73	2,038 ± 181	4,223 ± 345	338 ± 19	1,809 ± 160	171 ± 29
Taenia tecta, dorsal	1,151 ± 58	1,356 ± 124	1,878 ± 213	1,878 ± 213	2,977 ± 212	906 ± 64	3,662 ± 331	4,736 ± 428	308 ± 36	1,589 ± 141	357 ± 49
Taenia tecta, ventral	1,361 ± 86	1,022 ± 114	1,716 ± 125	1,716 ± 125	2,825 ± 222	788 ± 85	3,214 ± 272	4,316 ± 442	321 ± 45	1,377 ± 177	315 ± 90
Dorsal peduncular cortex	1,069 ± 62	1,624 ± 95	1,909 ± 255	1,909 ± 255	3,442 ± 155	1,048 ± 78	4,125 ± 340	5,048 ± 674	317 ± 52	1,031 ± 94	360 ± 44
Endopiriform nucleus (dorsal)	789 ± 51	1,480 ± 75	1,239 ± 129	1,239 ± 129	1,836 ± 224	695 ± 61	2,791 ± 257	4,059 ± 211	238 ± 23	1,032 ± 49	888 ± 95
Piriform cortex	1,060 ± 41	785 ± 48	1,821 ± 162	1,821 ± 162	3,328 ± 207	1,034 ± 68	3,961 ± 243	4,853 ± 158	448 ± 40	1,027 ± 80	242 ± 28
Entorhinal cortex, lateral	1,664 ± 59	1,054 ± 32	3,037 ± 224	3,037 ± 224	3,070 ± 198	1,297 ± 84	3,627 ± 221	5,894 ± 254	314 ± 19	1,771 ± 137	345 ± 26
Entorhinal cortex, medial	1,580 ± 102	1,040 ± 71	2,091 ± 301	2,091 ± 301	3,081 ± 268	1,190 ± 98	3,553 ± 487	6,201 ± 200	304 ± 26	1,770 ± 136	330 ± 17
Orbitofrontal cortex, medial	1,089 ± 83	1,095 ± 164	2,292 ± 278	2,292 ± 278	3,867 ± 360	1,553 ± 105	4,894 ± 323	5,925 ± 392	502 ± 38	650 ± 22	175 ± 43
Orbitofrontal cortex, ventrolateral	1,034 ± 79	995 ± 140	2,101 ± 236	2,101 ± 236	4,024 ± 511	1,520 ± 162	4,762 ± 406	5,909 ± 461	495 ± 48	570 ± 24	131 ± 28
Orbitofrontal cortex, lateral	1,100 ± 75	1,039 ± 175	2,168 ± 209	2,168 ± 209	3,983 ± 524	1,393 ± 172	4,269 ± 529	5,003 ± 404	513 ± 67	564 ± 22	211 ± 33
Olfactory tubercle	1,079 ± 88	964 ± 70	1,638 ± 131	1,638 ± 131	3,964 ± 346	853 ± 77	3,048 ± 403	2,995 ± 277	204 ± 25	770 ± 107	5,340 ± 439
Friedman ANOVA(χ^2^),	85.531***	78.060***	82.983***	82.983***	79.952***	93.630***	70.971***	94.811***	102.14***	105.714***	106.140***
***p <0.001											

*The Friedman ANOVAs display regional differences for each receptor type (all N = 10, df = 13). For pairwise comparisons between regions see further Supplementary Table 2*.

### Statistical Analysis

For statistical analysis, all measured values regarding our regions of interests were used. A Friedman ANOVA was performed across all subregions and layers for each receptor to detect general differences in the chemoarchitecture of the different areas of the olfactory system. If a significant result was obtained, a Wilcoxon rank test was then performed for pairwise inter-subarea comparisons (Supplementary Tables 4–16). A Dunn-Bonferroni *post-hoc* test was used for regional differences (Supplementary Table 2). Statistica 10 (StatSoft, Tulsa, RRID:SCR_015627) was used for Wilcoxon rank tests, SPSS (IBM Corp. Released 2017. IBM SPSS Statistics for Windows, Version 25.0. Armonk, NY: IBM Corp.) was used for the Friedman ANOVA and Dunn-Bonferroni *post-hoc* tests. The significance level was set to 0.05. Statistical data are available in Table 1, Supplementary Tables 2, 4–16.

A hierarchical cluster analysis was performed using SPSS Statistics 26 (**Figures 10**, **11**). The degree of similarity or dissimilarity between the multi-receptor balance of the analyzed areas was quantified in pairs over the Euclidean distance. The result was visualized in two dimensions using non-linear multidimensional scaling (MDS; **Figure 11**). The resulting clusters of the areas based on the receptor profile of the respective areas were determined by hierarchical cluster analysis (Ward linkage with Euclidean distances). To visualize the normalized mean values of the receptor densities and therefore to display the proportion of the individual receptors within a cluster, we generated a heat map. The order of the ROIs is based on the outcome of the cluster analysis and is represented by a dendrogram (**Figure 10**).

## Results

**Figure 2:**
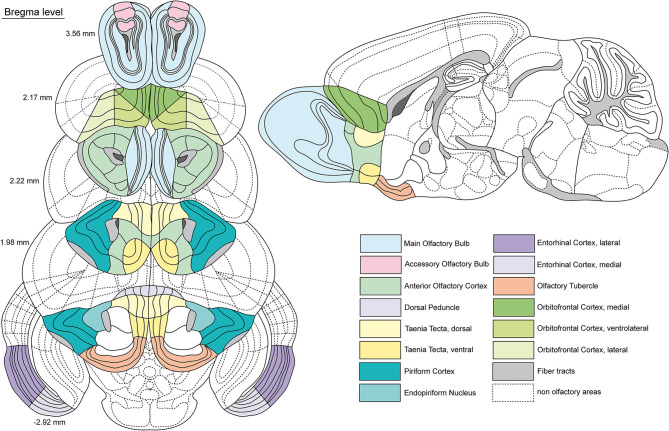
Schematic representation of the investigated olfactory areas. Coronal sections of the mouse (C57BL/6) brain along the anterior-posterior axis (related Bregma levels alongside). A sagittal section serves for further local orientation of certain areas. The investigated areas are indicated by different colors (see legend). Dashed lines show non olfactory areas for a better orientation. Gray areas show fiber tracts, whereas dark gray areas represent the subventricular zone. Boundaries adapted by the Allen Brain Reference Atlas (Lein et al., 2007; Dong, 2008).

**Figure 3:**
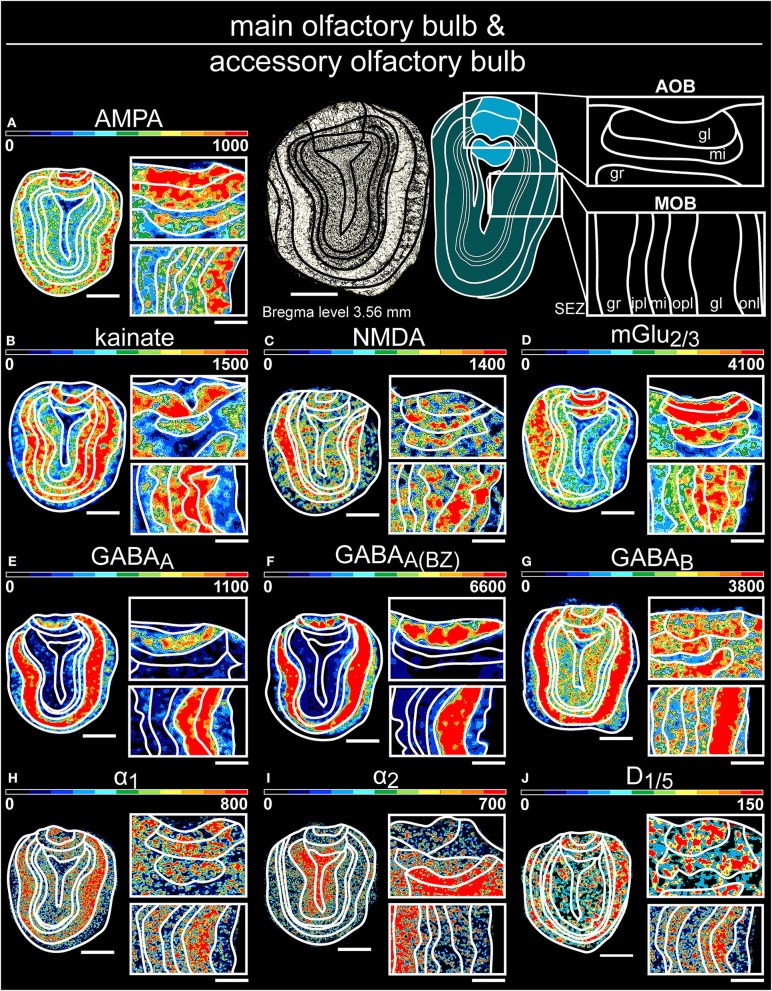
Color-coded autoradiographs showing the distribution and density of all investigated receptors of the main olfactory bulb **(A–J)** and accessory olfactory bulb **(K–T)**. Densities of the color-coded autoradiograms in fmol/mg protein according to color scales. For detailed receptor densities see Table 1. Scale bars: **(A–J)**, Coronal slice: 1.3 mm; Magnifications: 400 μm; onl, olfactory nerve layer; gl, glomerular layer; mi, mitral layer; ipl, inner plexiform layer; opl, outer plexiform layer; gr, granular layer; SEZ subependymal zone.

**Figure 4:**
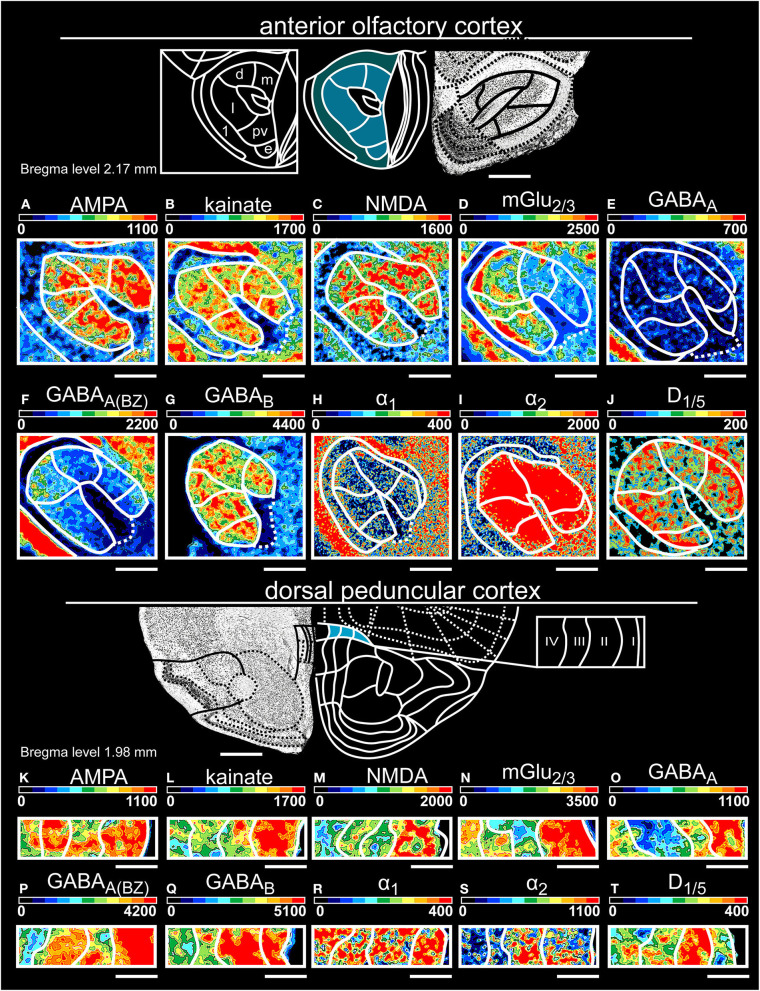
Color-coded autoradiographs showing the distribution and density of all investigated receptors of the anterior olfactory cortex **(A–J)** and dorsal peduncular cortex **(K–T)**. Densities of the color-coded autoradiograms in fmol/mg protein according to color scales. For detailed receptor densities see Table 1. Scale bars: Atlas, 800 μm; **(A–J)**, 600 μm; **(K–T)**, 200 μm; m, medial; d, dorsal; l, lateral; pv, posteroventral; 1, pars externa. I, layer 1; II, layer 2; III, layer 3; IV, layer 4.

**Figure 5:**
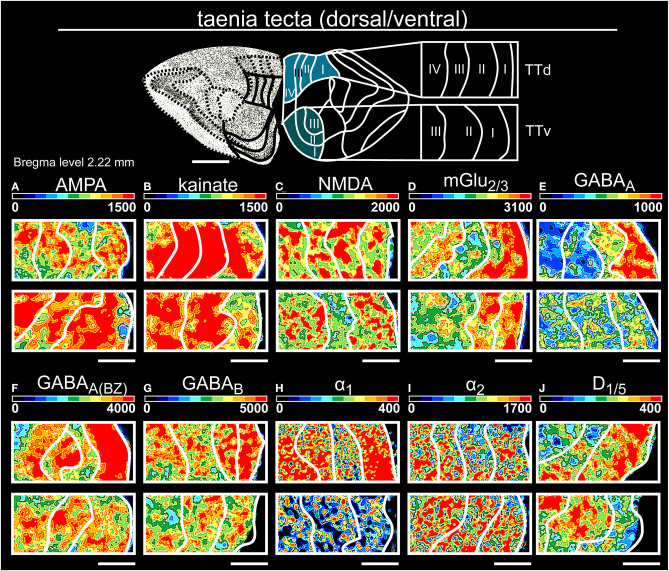
Color-coded autoradiographs showing the distribution and density of all investigated receptors of the taenia tecta (dorsal, ventral). Densities of the color-coded autoradiograms in fmol/mg protein according to color scales. For detailed receptor densities see Table 1. Scale bars: Atlas, 1 mm; **(A–J)**, 400 μm; TTd, taenia tecta, dorsal; TTv, taenia tecta, ventral. I, layer 1; II, layer 2; III, layer 3; IV, layer 4.

**Figure 6:**
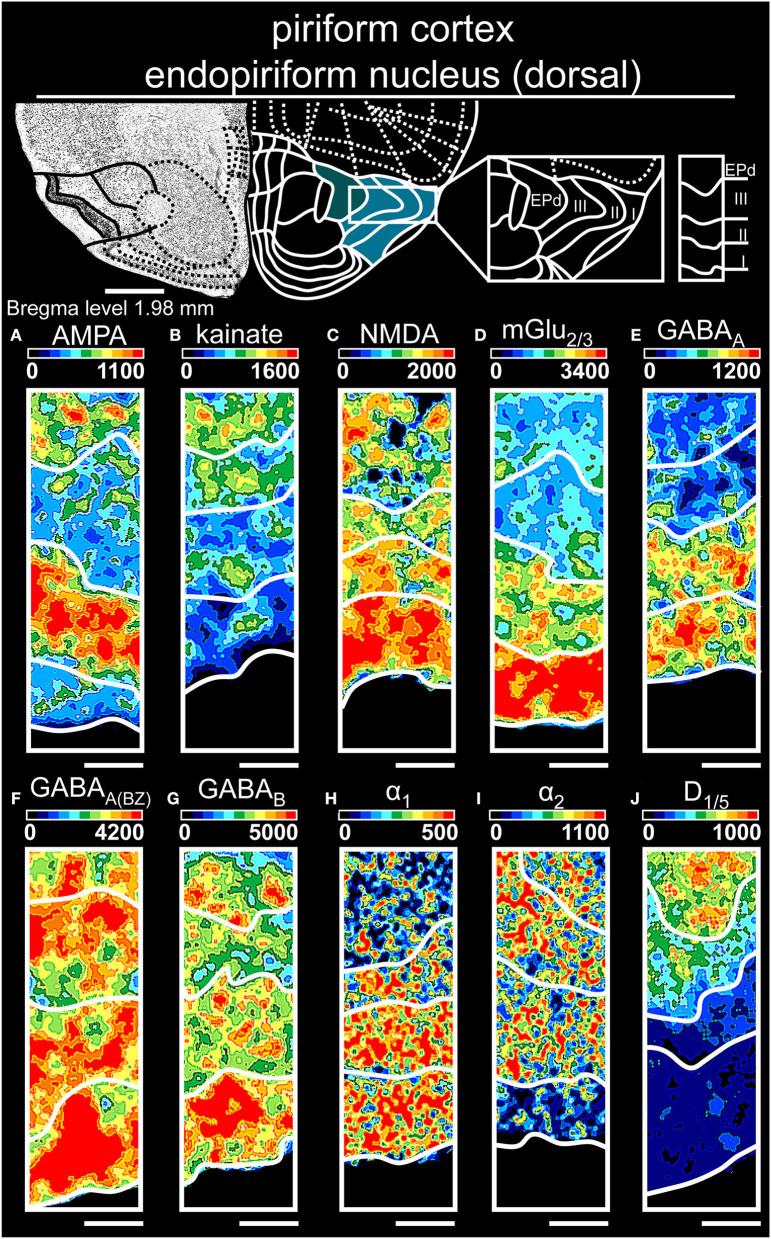
Color-coded autoradiographs showing the distribution and density of all investigated receptors of the piriform cortex and endopiriform nucleus (dorsal). Densities of the color-coded autoradiograms in fmol/mg protein according to color scales. For detailed receptor densities see Table 1. Scale bars: Atlas, 1.2 mm; **(A–J)**, 400 μm; EPd, dorsal endopiriform nucleus. I, layer 1; II, layer 2; III, layer 3.

**Figure 7:**
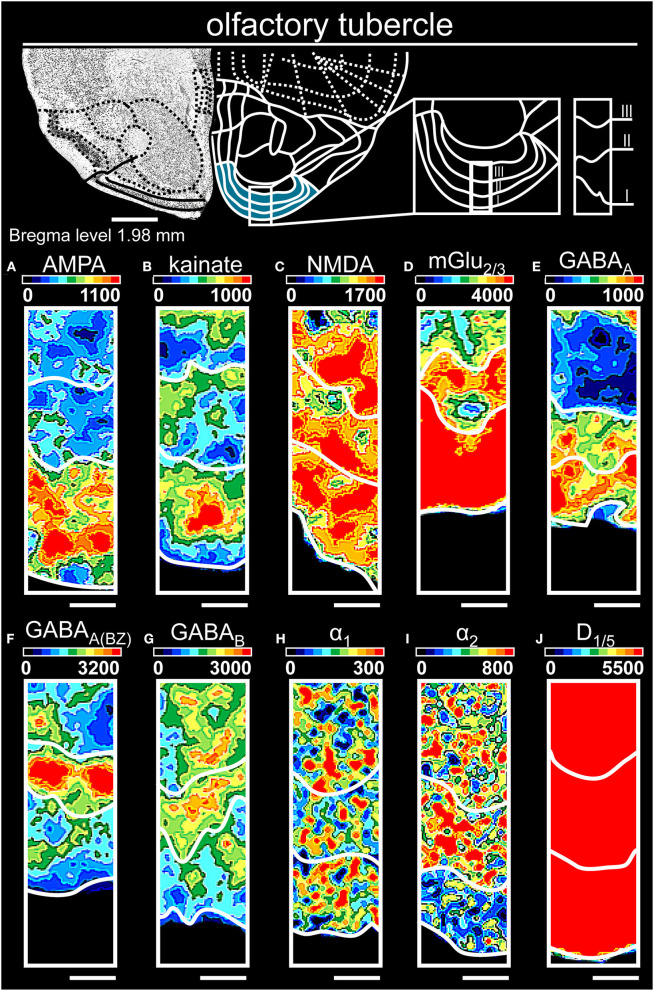
Color-coded autoradiographs showing the distribution and density of all investigated receptors of the olfactory tubercle. Densities of the color-coded autoradiograms in fmol/mg protein according to color scales. For detailed receptor densities see Table 1. Scale bars: Atlas, 1.2 mm; **(A–J)**, 400 μm. I, layer 1; II, layer 2; III, layer 3.

**Figure 8:**
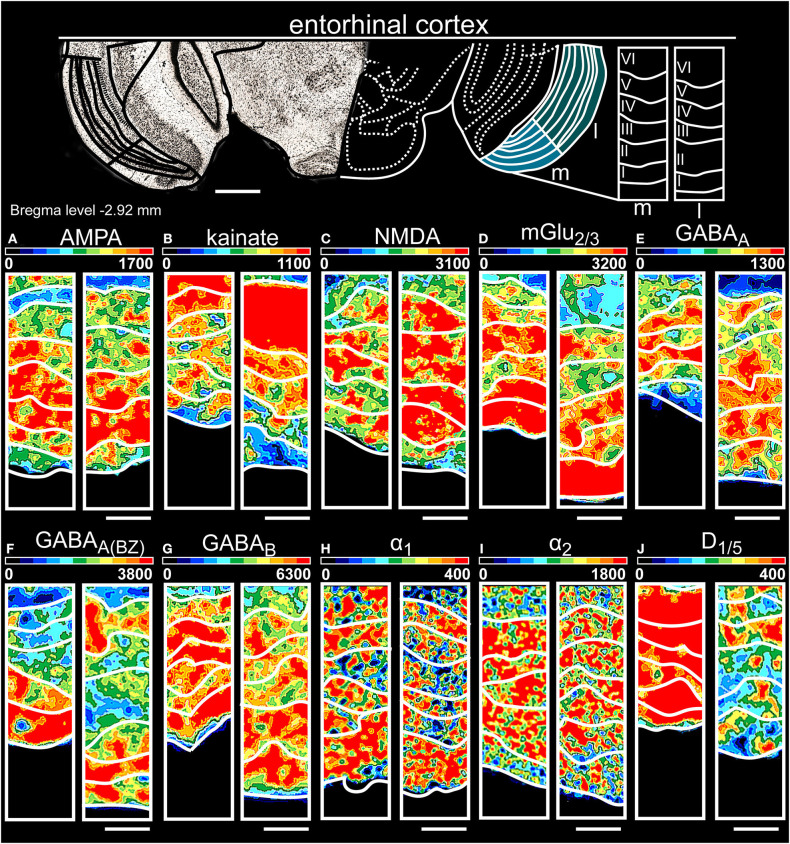
Color-coded autoradiographs showing the distribution and density of all investigated receptors of the entorhinal cortex (*l*, lateral; *m*, medial). Densities of the color-coded autoradiograms in fmol/mg protein according to color scales. For detailed receptor densities see Table 1. Scale bars: Atlas, 1.5 mm; **(A–J)**, 200 μm. I, layer 1; II, layer 2; III, layer 3; IV, layer 4; V, layer 5; VI, layer 6.

**Figure 9:**
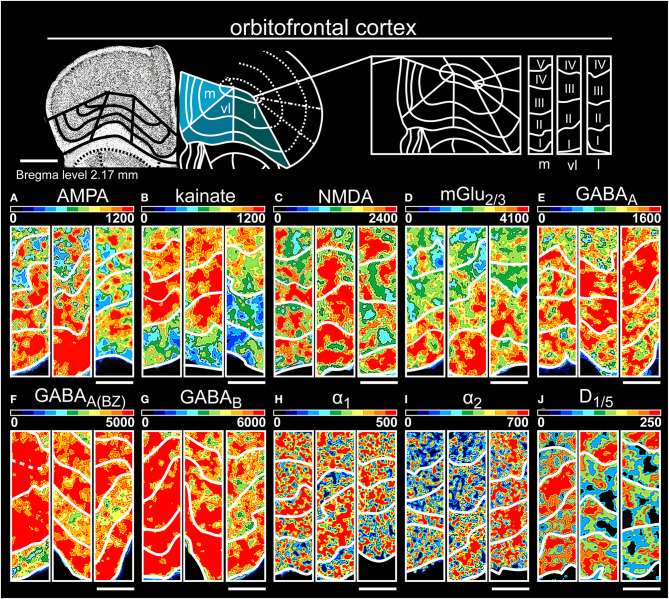
Color-coded autoradiographs showing the distribution and density of all investigated receptors of the orbitofrontal cortex (*l*, lateral; *m*, medial; *vl*, ventrolateral). Densities of the color-coded autoradiograms in fmol/mg protein according to color scales. For detailed receptor densities see Table 1. Scale bars: Atlas, 800 μm; **(A–J)**, 300 μm. I, layer 1; II, layer 2; III, layer 3; IV, layer 4; V, layer 5; VI, layer 6.

Areas of the main and accessory olfactory bulb at Bregma level 3.56 mm, the orbitofrontal cortex (medial, ventrolateral, lateral) and the anterior olfactory nucleus at Bregma level 2.17 mm, the piriform cortex, the taenia tecta (dorsal, ventral) at Bregma level 2.22 to 1.98 mm, the dorsal peduncular cortex at Bregma level 1.98 mm and the entorhinal cortex at Bregma level −2.92 mm have been mapped and analyzed. Figure 2 illustrates the anatomical subdivisions of the olfactory system that we used to map the receptor autoradiographs. The densities of the receptor binding sites of all analyzed receptors are presented for each region of interest in the color-coded autoradiographs (Figures 3–9), as well as in multireceptor fingerprints (**Figure 11**). The mean densities for each olfactory area are summarized in Table 1, the layer-specific densities are provided in Supplementary Table 2. All receptor binding densities are provided in fmol/mg protein. For statistical analyses see Table 1, Supplementary Tables 2, 4–16.

**Figure 10:**
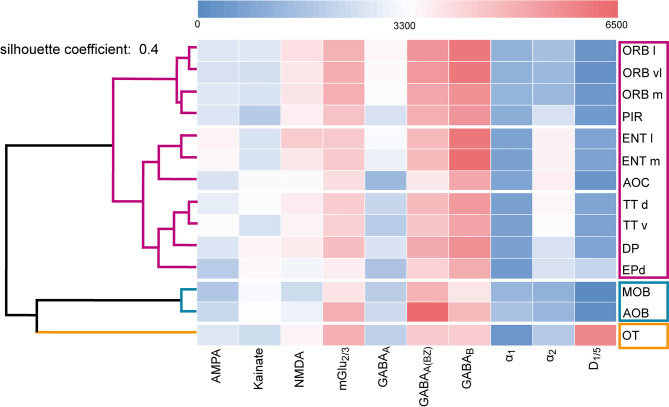
Dendrogram of the hierarchical cluster analysis. The area-specific receptor densities are displayed as a heat map according to their classification into clusters (Figure 11). The color legend represents the receptor densities (fmol/mg protein). Red color indicates high values (>3,300 fmol/mg protein), while blue color displays lower values (<3,300 fmol/mg protein). For abbreviations see (Figure 1).

**Figure 11:**
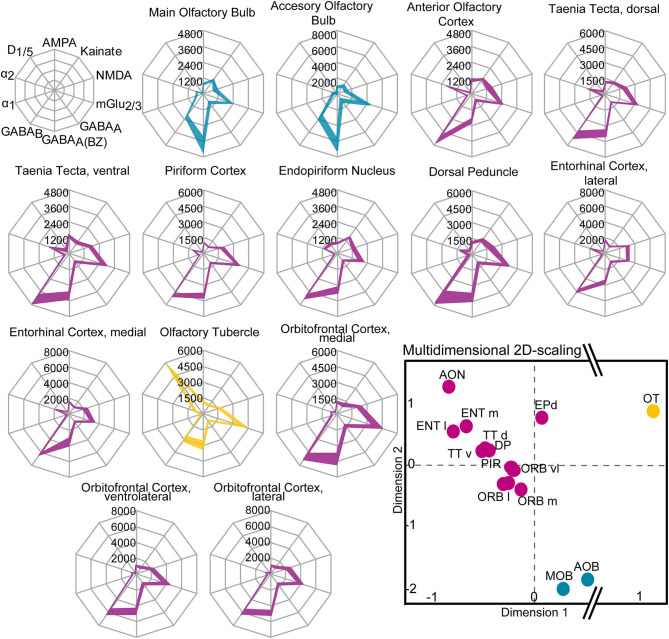
Receptor fingerprints of the 14 investigated olfactory areas. Mean densities (in fmol/mg protein) are provided for each individual receptor type, connected by a colored line (colors are similar to the multidimensional 2D-scaling analysis). The positions of the receptors are shown schematically in the first row. Filled areas mark upper and lower standard errors of the mean values of the receptor densities (± SEM). Lower right: Receptor-driven multidimensional 2D-scaling of the olfactory system. The dots represent a receptor feature vector, based on the area-specific multi-receptor balance of all investigated receptors (averaged over ten animals/hemispheres). The closer the dots, the smaller the Euclidean distance and the higher the similarity of the receptor architecture of the investigated areas. The hierarchical cluster analysis resulted in a three-cluster solution: the olfactory relay centers (blue), the cortical olfactory areas (violet) and the olfactory tubercle (yellow). The olfactory tubercle shows the highest Euclidean distance from all other areas (interruption of the X-axis, dimension 1). For abbreviations see (Figure 1).

Generally, all investigated receptors were differentially expressed in the olfactory system. Glutamatergic mGlu_2/3_Rs, and GABAergic benzodiazepine binding sites and GABA_B_Rs showed the highest densities, while low densities for α_1_Rs and D_1/5_Rs (except in the olfactory tubercle) were observed (see Figures 10, 11). In the following we will describe the highlights of different densities in the areas of the olfactory system.

### Receptor-Specific Differentiation of Olfactory Areas

#### Glutamatergic Receptors

Glutamatergic receptors revealed a heterogenous distribution in the olfactory system: mGlu_2/3_Rs were highly expressed from 1,836 ± 224 fmol/mg protein in the endopiriform nucleus up to 4,024 ± 511 fmol/mg protein in the ventrolateral orbitofrontal cortex (Supplementary Table 2). Compared to these densities, AMPARs, kainateRs and NMDARs showed lower densities (Table 1). AMPARs and kainateRs always differed in a homeostatic relationship in their densities, except in the orbitofrontal cortex. The highest concentration of AMPARs was observed in the lateral entorhinal cortex (1,664 ± 59 fmol/mg protein), while kainateRs showed the highest densities in the endopiriform nucleus (1,624 ± 95 fmol/mg protein). NMDARs showed high concentrations (up to 3,037 ± 224 fmol/mg protein for example in the lateral entorhinal cortex) except for the main olfactory bulb (954 ± 158 fmol/mg protein). mGlu_2/3_Rs showed high concentrations in the orbitofrontal cortex (4,024 ± 511 fmol/mg protein). The receptor marked the border of the accessory olfactory bulb (3,948 ± 376 fmol/mg protein) to the neighboring main olfactory bulb (2,180 ± 121 fmol/mg protein, *p* ≤ 0.001 Supplementary Table 2) and anterior olfactory cortex (2,365 fmol/mg protein, Table 1, *p* ≤ 0.025, Supplementary Table 2).

#### GABAergic Receptors

GABAergic receptors showed high concentrations in the olfactory system, compared to the other investigated receptors. GABA_A_Rs showed low receptor densities (803 ± 93 fmol/mg protein in the main olfactory bulb up to 1,553 ± 105 fmol/mg protein in the medial orbitofrontal cortex, Supplementary Table 2), while benzodiazepine binding sites showed higher concentrations (2,791 ± 257 fmol/mg protein up to 6,409 ± 664 fmol/mg protein in the accessory olfactory bulb, Table 1). Benzodiazepine binding sites were highest in the accessory olfactory bulb where they marked the border to the main olfactory bulb and the border of the anterior olfactory cortex to the ventral part of the taenia tecta (Supplementary Table 2). GABA_B_Rs showed the highest expression in the medial entorhinal cortex (6,201 ± 200 fmol/mg protein). The GABAR densities revealed differences between parts of the orbitofrontal cortex, particularly by low densities of GABA_B_Rs and GABA_A_Rs in the lateral part (Table 1, Supplementary Table 2).

#### Monoaminergic Receptors

Noradrenergic receptors revealed a heterogenic distribution. α_1_Rs showed comparatively low concentrations in the primary and secondary olfactory cortices from 513 ± 67 fmol/mg protein in the lateral orbitofrontal cortex up to 675 ± 50 fmol/mg protein in the accessory olfactory bulb. α_2_Rs showed higher densities in the olfactory cortices (up to 1,809 ± 160 fmol/mg protein) compared to the main olfactory bulb (497 fmol/mg protein, Supplementary Table 2). D_1/5_Rs showed low densities if compared to all investigated receptors. Only the olfactory tubercle revealed very high expression levels (5,340 ± 439 fmol/mg protein). This clearly marked the border of the olfactory tubercle to the neighboring piriform cortex (*p* ≤ 0.000, Supplementary Table 2).

### Layer-Specific Receptor Heterogeneity Within Olfactory Areas

The analysis of the individual receptors for each layer of an olfactory area further revealed a heterogeneous receptor profile for each area that is visualized schematically in Figures 3–9. Quantitative measurements of receptor densities (fmol/mg protein) for each area are provided in Table 1; Supplementary Table 3 with statistical analysis in Supplementary Tables 2, 4–18.

#### The Main and Accessory Olfactory Bulb

In general, all receptors, except AMPARs and α_2_Rs, were strongly expressed in the glomerular layer of the main olfactory bulb (Supplementary Tables 3, 4) and the mitral layer of the accessory olfactory bulb (Supplementary Tables 2, 3, 5). mGlu_2/3_Rs (Figure 3D), and GABA_B_Rs (Figure 3G) displayed significantly higher concentrations in the superficial layers of both while GABA_A(BZ)_Rs revealed the highest densities in the deeper layers of the main olfactory bulb (Figure 3F). In contrast, α_2_Rs and D_1/5_Rs were highest expressed in the granular layer of the accessory olfactory bulb (Figures 3I,J).

#### The Primary and Secondary Olfactory Cortices

In the anterior olfactory cortex (for layer-specific statistical analysis see Supplementary Table 6), all subareas showed high concentrations of GABA_B_Rs (Figure 4G) and mGlu_2/3_Rs (Figure 4D). Higher mGlu_2/3_, GABA_A(BZ)_ (Figure 4F) and α_1_ (Figure 4H) receptor densities separated the pars externa from the pars principalis. Here, higher densities of AMPA (Figure 4A) and GABA_B_ (Figure 4G) receptor densities characterized the medial part. The dorsal part of the anterior olfactory cortex was clearly separated by high concentrations of α_2_Rs (Figure 4I) and D_1/5_Rs (Figure 4J) and low densities of GABA_A(BZ)_Rs (Figure 4F). The posteroventral subarea showed the highest expression of kainateRs and the lowest densities of mGlu_2/3_Rs (Figure 4D) and α_1_Rs (Figure 4H). In the lateral part of the pars principalis, higher NMDA (Figure 4C) and GABA_B_ (Figure 4G) receptor densities and low concentrations of GABA_A_Rs (Figure 4E) separated this subarea from the remaining parts. The external subarea generally displayed lower expressions for all investigated receptors, except for GABA_A_Rs (Figure 4E).

The dorsal peduncular cortex (Table 1, Supplementary Tables 2, 3, 10) showed the lowest receptor concentrations in deep layer VI except for kainateRs (Figure 4L) and D_1/5_Rs (Figure 4T), which were highly expressed. GABAergic receptors revealed a high distribution in layer II/III and layer V.

Glutamatergic receptors were highly expressed in both areas of the taenia tecta (Table 1, Supplementary Tables 2, 3, 7, 8). Superficial layers I/II showed high concentrations of glutamatergic (except kainateRs, Figures 5A–D) and GABAergic receptors (Figures 5E,F) while catecholaminergic receptors were highest in deep layer IV (Figure 5I). GABA_B_Rs showed maximum densities in layer II of both areas but were significantly higher expressed in dorsal compared to ventral parts (Figure 5G).

The piriform cortex (Table 1, Supplementary Tables 2, 3, 11) revealed high densities of glutamatergic [NMDARs (Figure 6C), mGlu_2/3_Rs (Figure 6D)] and GABAergic receptors [GABA_A_Rs (Figure 6E), GABA_A(BZ)_Rs (Figure 6F)] in the superficial layer I, while deep layer III had highest concentrations of catecholaminergic receptors α_2_R (Figure 6I) and D_1/5_R (Figure 6J). The endopiriform nucleus (Table 1, Supplementary Table 3) showed high levels of GABAergic receptors (Figures 6E–G), while AMPARs (Figure 6A) and α_1_Rs (Figure 6H) revealed a low receptor distribution.

The entorhinal cortex (Table 1, Supplementary Tables 2, 3, 12, 13) expressed high levels of glutamatergic receptors [NMDA (Figure 8C), mGlu_2/3_Rs (Figure 8D)] and GABAergic receptors in layers II/III of both parts (Figures 8E–G). KainateRs (Figure 8B) and catecholaminergic receptors (Figures 8H–J) showed the highest expression in the deep layers V/VI. Although NMDARs (Figure 8C) and GABA_B_Rs (Figure 8G) were low expressed in layer VI of both subdivisions, their concentration in the medial part were higher compared to lateral. In general, layer II of both subdivisions showed the highest receptor densities.

The three areas of the orbitofrontal cortex (Table 1, Supplementary Tables 2, 3, 14–16) revealed high receptor densities in layers I/II for glutamatergic (Figures 9A–D) and GABAergic receptors (Figures 9E–G), except for kainateRs in layer VI (Figure 9B). While noradrenergic receptors were highest in layer III/V of the medial and lateral part, the ventrolateral part showed higher concentrations in layer I (Figures 9H,I). D_1/5_ receptors were generally low concentrated but highly expressed in deep layer VI (Figure 9J).

#### The Olfactory Tubercle

In the olfactory tubercle (Table 1; Supplementary Tables 2, 3, 9), low receptor concentrations (except mGlu_2/3_Rs [Figure 7D]) separated the molecular layer, while high AMPA (Figure 7A), NMDA (Figure 7C), GABA_A_ (Figure 7E) and D_1/5_ (Figure 7J) characterized the pyramidal layer. Noradrenergic (Figures 7H–J) and GABAergic (Figures 7E–G) receptors characterized the polymorphic layer.

### Molecular Structure of the Olfactory Subdivisions

Similarities and dissimilarities of the receptor architecture between the areas of the olfactory system were visualized in receptor fingerprints for each area (Figure 10). The receptor densities are averaged over all cortical layers or subdivisions representing the area-specific receptor balance of the ten investigated receptors (Table 1; Supplementary Table 2).

The fingerprints of the main and accessory olfactory bulb were highly similar in their shape. The same was true for areas of the primary and secondary olfactory cortices that differed mainly because of higher GABA_B_Rs, α_2_Rs, NMDARs, and lower benzodiazepine binding sites from the fingerprints of the main and accessory olfactory bulbs. The olfactory tubercle constituted its own cluster because of the high amount of D_1/5_Rs and mGlu_2/3_Rs. Additionally, the olfactory tubercle showed a low amount of GABA_A(BZ)_Rs and GABA_B_Rs, which was in contrast to the other olfactory areas, where GABA_B_Rs were higher concentrated compared to GABA_A(BZ)_Rs (Figure 11).

A multidimensional scaling analysis (Figure 11) and a dendrogram (Figure 10) showed a clear separation into three clusters (silhouette coefficient 0.4): (1) a cluster of the olfactory bulbs (main and accessory olfactory bulb), (2) a cluster of the areas of the primary and secondary cortex, excluding the olfactory tubercle (3) that constituted its own cluster. Based on the three-cluster solution, we visualized the influence of the individual receptors in a dendrogram and a heat map (Figure 10). For example, low α_2_Rs (blue color in heat map) and high GABA_A(BZ)_Rs (red color) distinguished the olfactory bulbs from the olfactory cortices. High D_1/5_Rs and low noradrenergic receptors distinguished the olfactory tubercle from the remaining subdivisions. The heat map showed a highly similar distribution of catecholaminergic receptors for the entorhinal cortex, anterior olfactory cortex and the taenia tecta (Figure 10).

## Discussion

Previous studies that focused on the receptors of the olfactory system were primarily related to the main olfactory bulb and the piriform cortex (for review see Shepherd, 2004; Ennis et al., 2007). Until now, the receptor architecture of the dorsal peduncular cortex, dorsal and ventral taenia tecta and the endopiriform nucleus has been scarcely analyzed. More generally, the identified olfactory areas have not been investigated as a comprehensive system yet. Therefore, there is no reliable basis for identifying alterations in the receptor balance in relation to disorders that are related to the olfactory system. Multiple receptor types play a role in neurodegenerative diseases (Armstrong et al., 1994; Hawkes, 2006; Zhang et al., 2015; Kwakowsky et al., 2018) and dysfunctions (Thompson et al., 2006; Yuan and Slotnick, 2014; Münster et al., 2020). Thus, the area- and layer-specific receptor balance of the entire olfactory system provides valuable pharmacological targeting-indications in case of disease-related alterations of the investigated receptor distribution patterns. For example, a bilateral bulbectomy and thus, a change in the neurotransmitter system is capable to induce the symptoms of major depression (Song and Leonard, 2005; Yuan and Slotnick, 2014) and α_2C_ adrenoceptors were identified as a potential pharmacological target for neurodegenerative and neuropsychiatric disorders such as depression and schizophrenia (Arponen et al., 2014). The study of Apronen and colleagues suspected the highest α_2C_ receptor concentration in the human olfactory tubercle and striatum, whereas our data and other studies detected the highest expression in the rodent anterior olfactory cortex and the entorhinal cortex (Scheinin et al., 1994; Holmberg et al., 2003).

The receptor fingerprint of an area provides an indication of its functional features (Zilles et al., 2002a, 2015; Eickhoff et al., 2008; Palomero-Gallagher et al., 2009; Zilles and Palomero-Gallagher, 2017; Impieri et al., 2019). Furthermore, the similarity of the fingerprints allows inferences regarding a common network (Palomero-Gallagher et al., 2009; Zilles et al., 2015; Palomero-Gallagher and Zilles, 2018).

### The Special Role of the Olfactory Tubercle in the Olfactory System

The olfactory tubercle builts a single cluster due to its high amounts of dopaminergic receptors, which was also reported in earlier studies (Wamsley et al., 1991; Duffy et al., 2000). Usually, dopaminergic receptors become active following the perception of reward promising olfactory signals, explaining the role of the olfactory tubercle as a motivational and evaluating area for olfactory preferences (Ikemoto, 2007; Zhang et al., 2017; Murata et al., 2019). The neurochemical structure of the olfactory tubercle has been extensively researched (Cansler et al., 2020). Its unique position in the olfactory system is caused by the fact that the olfactory tubercle is considered to be part of the olfactory cortex as well as the ventral striatum (de Olmos and Heimer, 1999; Cansler et al., 2020). Because of the high dopamine receptor density, the olfactory tubercle displays a receptor profile, which is more similar to the striatal than to the olfactory system (Knable et al., 1994; Sulzer et al., 2016). However, further autoradiographic studies of the striatum would be necessary to gain more comparisons at this point. In general, more attention should be paid on the olfactory tubercle as a multisensory region, as it seems to play a crucial role in odor-guided behavior (Fitzgerald et al., 2014; Murata et al., 2015; Murata, 2020). The olfactory tubercle also differentiates in its cytoarchitecture, as it contains clusters of granule cells (Islands of Calleja) as well as a trilaminar cortical organization (Pigache, 1970). These cells are divided into different groups and are located in layers II and III of the olfactory tubercle and receive input from the main olfactory bulb (Bayer, 1985; Xiong and Wesson, 2016). The receptor architecture indicates that layer I has significantly lower receptor concentrations than layer II/III. Only mGlu_2/3_Rs are higher expressed in layer I which is correlated with a direct input from the tufted cells of the main olfactory bulb (Scott et al., 1980; Imamura et al., 2011; Xiong and Wesson, 2016). The olfactory tubercle also differs from the olfactory cortex in embryogenesis and shows more similarities to the ventral striatum (Bayer, 1985). For example, ventricular zone progenitors in the subpallium give rise to astrocytes in the ventral striatum and the olfactory tubercle (Torigoe et al., 2015). The olfactory tubercle and the piriform cortex develop their laminae prior to the olfactory cortex (Schwob and Price, 1984a,b), suggesting that both areas have advanced maturation and function at an early age (Wesson and Wilson, 2011).

### The Molecular Organization in the Olfactory Cortex in Comparison to Function

The hierarchical cluster analysis of the receptor fingerprints revealed a three-cluster-solution. The main and accessory olfactory bulb were very similar in their receptor balance. They constitute a cluster and show greater distance in their similarity to the primary and secondary olfactory cortex, because both areas serve the primary acquisition and processing of olfactory information (Mucignat-Caretta, 2010; Cleland and Linster, 2019).

The primary and secondary olfactory cortices clustered as olfactory processing areas, consisting of three smaller clusters. First, the taenia tecta, the dorsal peduncular cortex and the endopiriform nucleus clustered. The functional role of these areas as part of the olfactory system has not been fully investigated yet. Both, the taenia tecta and the dorsal peduncular cortex showed a highly heterogeneous receptor profile and differ mainly in their densities of glutamatergic receptors and α_2_Rs from the other analyzed areas. Until now, only group II mGluRs (McOmish et al., 2016), subunits of GABA_A_Rs (Zhang et al., 1991) and D_1_Rs in pyramidal and GABAergic neurons of the taenia tecta (Santana et al., 2009) were analyzed. The knowledge about these areas is incomplete, however, they may play an important role in essential olfactory functions. The taenia tecta and the dorsal peduncular cortex participate in associative and extinction learning and exhibit connections to the lateral entorhinal cortex, piriform cortex, main olfactory bulb and the olfactory tubercle (Haberly and Price, 1978; Ottersen, 1982; Wyss and Sripanidkulchai, 1983; Santiago and Shammah-Lagnado, 2005; Peters et al., 2009; Cleland and Linster, 2019). It is noticeable that increased concentrations of kainateRs contributed to the clustering, while kainateRs are well-known for their crucial role in epilepsy (Falcón-Moya et al., 2018). However, future studies should focus on the functional network of these clustering areas. The dorsal endopiriform nucleus is located next to the cluster of the taenia tecta and the dorsal peduncular cortex. Its function also remains unclear, but previous studies assumed that the dorsal endopiriform nucleus integrates olfactory processed information of the piriform cortex with gustatory information of the gustatory cortex (Sugai et al., 2012). Similar to the piriform cortex, it is highly epileptogenic (Hoffman and Haberly, 1993, 1996; Demir et al., 1998) but differs significantly in its receptor architecture from the piriform cortex. Presumably, because the dorsal endopiriform nucleus is closely connected to the claustrum and the insular cortex and is thus also involved in the processing of non-olfactory information (Sugai et al., 2012). This discrepancy may constitute an important criterion in the study of epilepsy. In general, the dorsal endopiriform nucleus and the claustrum together are considered to be the claustral complex. Both regions derive from the lateral pallium and play a crucial role in limbic circuitry (Watson and Puelles, 2017; Bruguier et al., 2020).

Second, the entorhinal cortex clustered with the anterior olfactory cortex. The clustering of the entorhinal and anterior olfactory cortex is particularly interesting because both areas play a key role during the development of neurodegenerative diseases. The entorhinal cortex, which belongs to the hippocampal formation, connects the hippocampal formation with the olfactory system (Haberly and Price, 1978; Stäubli et al., 1984; Chapuis et al., 2013; Leitner et al., 2016), including the anterior olfactory cortex (Luskin and Price, 1983; Wyss and Sripanidkulchai, 1983; Mason et al., 2016; Cleland and Linster, 2019). Here, receptors play a decisive role in Alzheimer's disease (Armstrong et al., 1994; Kwakowsky et al., 2018; Berggaard et al., 2019) and also epilepsy (Spencer and Spencer, 1994; Du et al., 1995; Mann et al., 2009; Nibber et al., 2017; Stefanits et al., 2019). Early studies discovered neurofibrillary tangles and neuritic plaques in combination with cell loss limited to the anterior olfactory cortex in patients with Alzheimer's disease (Esiri and Wilcock, 1984; Ohm and Braak, 1987; Ubeda-Bañon et al., 2020). Further, the pathological dysfunctionality of the human entorhinal cortex causes impairment of memory function in Alzheimer's disease (Van Hoesen et al., 1986; Braak and Braak, 1991). In Parkinson's disease, the anterior olfactory cortex plays a key role for olfactory dysfunctions, because of neuronal depletion and the occurrence of Lewy Bodies in early stages (Pearce et al., 1995; Braak et al., 2003; Beach et al., 2009; Mason et al., 2016). Thus, the anterior olfactory cortex and the entorhinal cortex constitute key components of the olfactory system in both, Alzheimer's and Parkinson's disease, and are linked to network dysfunction in these diseases (Ubeda-Bañon et al., 2020). Here, our data indicate a correlation of both areas at the receptor level and offer a further approach to examine these diseases.

Third, the orbitofrontal cortex clustered with the piriform cortex. Both areas are involved in olfactory discrimination (Staubli et al., 1987; Critchley and Rolls, 1996; Schoenbaum et al., 1999; Lazic et al., 2007). Furthermore, it was discovered that the projections between the piriform cortex and the orbitofrontal cortex serve as a motivation-related pathway in opioid abstinence (Reiner et al., 2020), since the piriform cortex has reciprocal connections to the orbitofrontal cortex and the amygdaloid complex (Illig, 2006). Additionally, atrophies in the piriform cortex and orbitofrontal cortex were also observed in early stages of Parkinson‘s disease and contribute to olfactory dysfunctions (Lee et al., 2020). However, further research is required to establish the full extent of the impact at an organizational level (Li et al., 2010; Xu et al., 2012; Cremer et al., 2015a,b; Zhang et al., 2015; Perez-Lloret and Barrantes, 2016; Kwakowsky et al., 2018).

### Comparison of the Layer-Specific Organization of the Olfactory System

Up to now, few studies have investigated receptor densities of the individual layers of the olfactory areas. In the main olfactory bulb, we observed high expression rates of GABA_A(BZ)_Rs in the olfactory nerve layer, which is in line with a study that detected high levels of [^3^H]Ro5-4864 binding sites, a ligand for selective peripheral-type benzodiazepine receptors (Anholt et al., 1984). Our data are consistent with other studies of the piriform cortex, that detected the highest concentrations of AMPARs in layer II (Petralia and Wenthold, 1992), high levels of NMDARs (Petralia et al., 1994b) and mGlu_2/3_Rs (Wada et al., 1998) in layers I/II and kainateRs (Wisden and Seeburg, 1993; Petralia et al., 1994a) in layer III. We can also confirm that GABA_A_Rs are mainly distributed in layers I and II (Ennis et al., 2007). In line with earlier reports, we further observed the minimum receptor density of α_1_Rs (Jones et al., 1985) and high concentrations of α_2_Rs (Unnerstall et al., 1984) in layer III of the piriform cortex.

GABA_A_Rs and GABA_B_Rs are present in the anterior olfactory cortex (Bowery et al., 1987; Zhang et al., 1991), but our data showed a heterogenous distribution of the receptors: GABA_B_Rs are significantly higher expressed compared to GABA_A_Rs. High densities of GABA_A(BZ)_Rs were also observed in the anterior olfactory cortex of the turtle (Schlegel and Kriegstein, 1987) and rat (Richards et al., 1987). Receptors for noradrenaline and dopamine were observed in the anterior olfactory cortex (Fallon and Moore, 1978), but we found significant differences in the subareas with respect to the receptor distribution. While α_1_Rs were highly concentrated in the pars externa, high levels of α_2_Rs were observed in the pars principalis, particularly dorsal, medial and posteroventral (Unnerstall et al., 1984). In line with our observations for D_1/5_Rs, Savasta and colleagues found high amounts of D_1_Rs in the rat anterior olfactory cortex (Savasta et al., 1986). Particularly in the anterior olfactory cortex, significant differences in receptor densities were observed in the dorsal part. While GABA_A_Rs, α_1_Rs and D_1/5_Rs showed low amounts, mGlu_2/3_Rs, α_2_Rs and GABA_B_Rs were present in high concentrations.

In the lateral entorhinal cortex, a strong expression of group II and III mGluRs was detected (Ohishi et al., 1993; Fotuhi et al., 1994; Wright et al., 2013). Here, we observed the highest expression of mGlu_2/3_Rs in layer II and III.

Up to now only few receptor data exist for the olfactory tubercle. KainateRs were investigated in previous studies (Gall et al., 1990; Bischoff et al., 1997), but no conclusions were made regarding the layer specificity. We observed highest levels of kainateRs and GABA_A(BZ)_Rs in the polymorphic layer. High concentrations of GABA_A(BZ)_Rs were detected in GABAergic receptors using flunitrazepam (Biscoe et al., 1984). In line with our results, α_2_Rs occur in the plexiform and polymorphic layers (Unnerstall et al., 1984). We further confirmed a strong expression of D_1_Rs (Wamsley et al., 1991), particularly D_1/5_Rs (Duffy et al., 2000) in the pyramidal layer of the olfactory tubercle.

## Conclusion

The present study provides a detailed layer-specific multireceptor-architectonic characterization of the mouse olfactory system. The clustering leads to the conclusion that the chemical organization of olfactory tubercle is clearly different from the remaining olfactory system, while the secondary and primary olfactory cortex build three distinct clusters. This leads to novel coherences in connectivity, for example for the close relationship between the taenia tecta, the dorsal peduncular cortex and the dorsal endopiriform nucleus. Furthermore, our results provide comparative reference data for future studies in the human olfactory system. Considering that the human olfactory system is affected in the first instance in multiple neurodegenerative diseases and dysfunctions, our data may provide new study approaches regarding pharmacological targeting research and external and internal determinants, including cognitive training and adult neurogenesis. Additionally, the resulting molecular atlas can be used for comparative studies to parcellate the olfactory system in other mammals like primates to gain further functional and molecular insights.

## Data Availability Statement

The original contributions presented in the study are included in the article/Supplementary Material, further inquiries can be directed to the corresponding author/s.

## Ethics Statement

The animal study was reviewed and approved by Landesamt für Natur, Umwelt und Verbraucherschutz NRW, Germany (LANUV) National Institute of Health Guide for Care and Use of Laboratory Animals.

## Author Contributions

KL performed the analysis of the receptor autoradiograms, designed the figures, evaluated the results and wrote the initial draft of the manuscript. KA provided critical feedback and expertise for the manuscript. CH designed the study, verified the methods and supervised the project. KA and CH acquisitioned funding for the project. All authors reviewed and edited the manuscript during each stage and approved the final manuscript.

## Conflict of Interest

The authors declare that the research was conducted in the absence of any commercial or financial relationships that could be construed as a potential conflict of interest.
