# Supplementary material for: The Neurotransmitter Receptor Architecture of the Mouse Olfactory System
Source: Front Neuroanat. 2021 Apr 23;15:632549. doi: 10.3389/fnana.2021.632549 (PMC8102831; doi:10.3389/fnana.2021.632549)
Supplement: Supplementary file 1 [file Table_1.DOCX]

Supplementary Material

**Supp. Tab. 1**: Incubation conditions used for receptor autoradiography, including the 3H-ligand and displacer for glutamatergic (AMPA, kainite, NMDA, mGluR_2/3_), GABAergic (GABA_A_, GABA_A(BZ)_, GABA_B_), and catecholaminergic (α_1_, α_2_, D_1/5_) receptors.

| Receptor | [^3^H] ligand (incubation concentration) | Displacer (incubation concentration) | Incubation buffer | Preincubation step | Main incubation step | Rinsing step |
| --- | --- | --- | --- | --- | --- | --- |
| AMPA | [^3^H] AMPA (10 nM) | Quisqualate (10 µM) | 50 nM Tris-acetate (pH 7.2) | 3 x 10 min at 4°C in incubation buffer | 45 min at 4°C in  incubation buffer  + 100 mM KSCN | 4 x 4 sec at 4 °C in  incubation buffer  + 2 x 2 sec at 25°C in  acetone/glutaraldehyde |
| Kainate | [^3^H] Kainate (9.4 nM) | SYM-2081 (100 µM) | 50 nM Tris-citrate (pH 7.1) | 3 x 10 min at 4°C in incubation buffer | 45 min at 4°C in incubation buffer + 10 nM Ca-acetate | 3 x 4 sec at 4 °C in  incubation buffer  + 2 x 2 sec at 25°C in  acetone/glutaraldehyde |
| NMDA | [^3^H] MK-801 (3.3 nM) | MK-801 (100 µM) | 50 nM Tris-HCl (pH 7.2) | 15 min at 4°C in incubation buffer | 60 min at 25°C in incubation buffer  + 50 μM glutamate  + 30 μM glycine  + 50 μM spermidine | 2 x 5 min at 4°C in  incubation buffer  + 50 μM glutamate  + 1 dip at 25°C in H_2_O dest. |
| mGluR_2/3_ | [^3^H] LY-341495 (1 nM) | L-glutamate (1 mM) | 10 nM phosphate buffer (pH 7.6) | 2 x 5 min at 4°C in incubation buffer | 60 min at 4°C in  incubation buffer  + 100 mM KBr | 2 x 5 min at 4°C in  incubation buffer  + 1 dip at 25°C in H_2_O dest |
| GABA_A_ | [^3^H] muscimol (7.7 nM) | GABA (10 µM) | 50 nM Tris-citrate (pH 7.0) | 3 x 5 min at 4°C in incubation buffer | 40 min at 4°C in  incubation buffer | 3 x 3 min at 4°C in  incubation buffer  + 1 dip at 25°C in H_2_O dest |
| GABA_B_ | [^3^H] CGP-54626 (2 nM) | CGP-55845 (100 µM) | 50 nM Tris-HCl (pH 7.2) | 3 x 5 min at 4°C in incubation buffer | 60 min at 4°C in  incubation buffer  + 2,5 mM CaCl_2_ | 3 x 2 min at 4°C in  incubation buffer  + 1 dip at 25°C in H_2_O dest |
| GABA_BZ_ | [^3^H] Flumazenil (1 nM) | Clonazepam (2 µM) | 170 nM Tris-HCl (pH 7.4) | 15 min at 4°C in incubation buffer | 60 min at 4°C in  incubation buffer | 2 x 1 min at 4 °C in incubation buffer  + 1 dip in H_2_O dest |
| α_1_ | [^3^H] Prazosin (0.09 nM) | Phentolamine (10 µm) | 50 mM Na/K phosphate buffer (pH 7.4) | 15 min at 25°C in incubation buffer | 60 min at 25°C in incubation buffer | 2 x 5 min at 4°C in incubation buffer  + 1 dip at 25°C in H_2_O dest |
| α_2_ | [^3^H] RX 821002 (1.4 nM) | Phentolamine (10 µm) | 50 nM Tris-HCl (pH 7.7) | 15 min at 25°C in incubation buffer | 90 min at 25°C in incubation buffer | 5 min at 4°C in incubation buffer  + 1 dip at 25°C in H_2_O dest |
| D_1/5_ | [^3^H] SCH-23390 (1.67 nM) | SKF-83566 (1 µM) | 50 nM Tris-HCl (pH 7.4) | 20 min at 25°C in incubation buffer  + 120 mM NaCl  + 5 mM KCl  + 2 mM CaCl_2_  + 1 mM MgCl_2_ | 90 min at 25°C in incubation buffer  + 120 mM NaCl  + 5 mM KCl  + 2 mM CaCl_2_  + 1 mM MgCl_2_  + 1 μM Mianserine | 6 x 1 min at 4°C in  incubation buffer  + 0,1 % Ascorbic acid  + 150 nM NaCl  + 1 dip in H_2_O dest. |
